# Pathophysiological Implications of Urinary Peptides in Hepatocellular Carcinoma

**DOI:** 10.3390/cancers13153786

**Published:** 2021-07-27

**Authors:** Ayman Bannaga, Jochen Metzger, Torsten Voigtländer, Martin Pejchinovski, Maria Frantzi, Thorsten Book, Sean James, Kishore Gopalakrishnan, Harald Mischak, Michael P. Manns, Ramesh P. Arasaradnam

**Affiliations:** 1 Department of Gastroenterology and Hepatology, University Hospital, Coventry CV2 2DX, UK; r.arasaradnam@warwick.ac.uk; 2 Warwick Medical School, University of Warwick, Coventry CV4 7HL, UK; 3 Mosaiques Diagnostics GmbH, 30659 Hannover, Germany; metzger@mosaiques-diagnostics.com (J.M.); pejchinovski@mosaiques-diagnostics.com (M.P.); frantzi@mosaiques-diagnostics.com (M.F.); mischak@mosaiques-diagnostics.com (H.M.); 4 Department of Gastroenterology, Hepatology and Endocrinology, Hannover Medical School, 30625 Hannover, Germany; voigtlaender.torsten@mh-hannover.de (T.V.); book.thorsten@mh-hannover.de (T.B.); manns.michael@mh-hannover.de (M.P.M.); 5 Arden Tissue Bank, University Hospital, Coventry CV2 2DX, UK; sean.james@uhcw.nhs.uk; 6 Department of Pathology, University Hospital, Coventry CV2 2DX, UK; kishore.gopalakrishnan@uhcw.nhs.uk; 7 Faculty of Health & Life Sciences, Coventry University, Coventry CV1 5FB, UK; 8 Leicester Cancer Research Centre, University of Leicester, Leicester LE1 7RH, UK

**Keywords:** hepatocellular carcinoma, capillary electrophoresis mass spectrometry, urinary peptides, diagnosis, prognosis

## Abstract

**Simple Summary:**

In this study, the application of capillary electrophoresis mass spectrometry enabled identification of 31 urinary peptides significantly associated with hepatocellular carcinoma diagnosis and prognosis. Further assessment of these peptides lead to prediction of cellular proteases involved in their development namely Meprin A subunit α and Kallikrein-6. Subsequent identification of the proteases was verified by immunohistochemistry in normal liver, cirrhosis and hepatocellular carcinoma. Histopathological assessment of the proteases revealed numerical gradient staining signifying their involvement in liver fibrosis and hepatocellular carcinoma formation. The discovered urinary peptides offered a potential noninvasive tool for diagnosis and prognosis of hepatocellular carcinoma.

**Abstract:**

Hepatocellular carcinoma (HCC) is known to be associated with protein alterations and extracellular fibrous deposition. We investigated the urinary proteomic profiles of HCC patients in this prospective cross sectional multicentre study. 195 patients were recruited from the UK (Coventry) and Germany (Hannover) between 1 January 2013 and 30 June 2019. Out of these, 57 were HCC patients with a background of liver cirrhosis (LC) and 138 were non-HCC controls; 72 patients with LC, 57 with non-cirrhotic liver disease and 9 with normal liver function. Analysis of the urine samples was performed by capillary electrophoresis (CE) coupled to mass spectrometry (MS). Peptide sequences were obtained and 31 specific peptide markers for HCC were identified and further integrated into a multivariate classification model. The peptide model demonstrated 79.5% sensitivity and 85.1% specificity (95% CI: 0.81–0.93, *p* < 0.0001) for HCC and 4.1-fold increased risk of death (95% CI: 1.7–9.8, *p* = 0.0005). Proteases potentially involved in HCC progression were mapped to the N- and C-terminal sequence motifs of the CE-MS peptide markers. In silico protease prediction revealed that kallikrein-6 (KLK6) elicits increased activity, whilst Meprin A subunit α (MEP1A) has reduced activity in HCC compared to the controls. Tissue expression of KLK6 and MEP1A was subsequently verified by immunohistochemistry.

## 1. Introduction

Hepatocellular carcinoma (HCC) incidence is increasing worldwide, and it is the third most frequent cause of cancer related death globally [1]. HCC is more frequent in males than females and usually occurs at older ages (>60 years). Most patients who develop HCC are asymptomatic in the early stages of disease, with features of abdominal pain, abdominal mass and deranged liver function tests (LFTs) infrequently present. HCC is typically identified clinically when patients affected by liver cirrhosis (LC) develop sudden hepatic decompensation with features such as ascites, jaundice, hepatic encephalopathy, or variceal bleeding [2,3,4].

HCC is the end result of progressive liver fibrosis and liver cirrhosis (LC). Various causes can lead to chronic liver injury provoking an inflammatory response and resulting in liver fibrosis through activation of the hepatic stellate cells. At the molecular level, this activation is associated with protein changes in the liver extracellular matrix (ECM). The ECM consists of an array of various proteins that comprise the scaffolding of the liver. Morphologically liver fibrosis is characterized by an excessive deposition of collagen-rich ECM components [5].

For many years, 2D gel electrophoresis was the principal proteomic technology. It is now largely replaced by mass spectrometry detection usually connected to a preceding non-gel-based separation through liquid chromatography (LC) or capillary electrophoresis (CE) enabling multidimensional analyte detection in complex biofluids with high-resolution capacity. Due to these characteristics, mass spectrometry-based techniques are increasingly used in medical research including proteomic characterization, biomarker identification and diagnostic evaluation of liver and other tumours [6,7,8,9]. Capillary electrophoresis mass spectrometry (CE-MS) has emerged in recent years as a hybrid technology using capillary electrophoresis (CE) instead of liquid chromatography for sensitive (up to 1 fmol) and high-resolution low molecular weight protein and peptide separation before mass spectrometry (MS). CE-MS does not require a sieving matrix, and it also does not depend on buffer gradients and, since no continuous adaptation of electrospray conditions is needed for optimal ionization, separation and detection of samples can be conducted fully automated. Clinical application of the CE-MS system used in this study has been demonstrated in technical reports and previous large-scale clinical studies [10,11,12,13]. Notably, this method enables profiling of the proteomic content of body fluids, such as urine, plasma or bile, in a mass range of 0.8 to 20 kilodalton (kDa). So far, it is one of the most applicable methods for monitoring of systemic catabolic processes caused by differences in the proteolytical environment at tissue and organ sites [14,15,16].

In this multicentre study, we applied the CE-MS technology to investigate the low molecular weight proteome of urine from patients with HCC and non-HCC but with various liver diseases including non-alcoholic fatty liver disease (NAFLD), non-alcoholic steatohepatitis (NASH) and liver cirrhosis (LC). The purpose of this study was to identify peptide markers specific for HCC, as currently there are none suitable in clinical practice. Urine as a biological medium is easy to collect and will be better accepted as an investigative tool for HCC.

Considering the latest advancements in machine learning, the aim was to integrate the markers in a multivariate classification model. Apart from combining these peptide markers to a multivariate pattern to support HCC diagnosis, we additionally investigated the origin of these peptide fragments by resolving their amino acid sequence and by searching for proteases involved in their generation through the in silico mapping software tool Proteasix [17]. This tool enables the linking of peptide fragments to active proteases and is therefore the bridge between the phenotype depicted in the low molecular weight proteome (consisting of naturally occurring peptides as a result of proteolysis) and the protease activity as a result of molecular pathophysiological mechanisms that are altered in diseases. This online open-source tool uses an input peptide list and allows for automatic cleavage site reconstruction and protease associations based on N- and C- terminus mapping. In this study, in silico prediction of potential proteases was performed as proof for their involvement in peptide cleavage that occurs in HCC pathogenesis at the tumour site. The mapped proteases were additionally evaluated for differences in their proteomic expression by immunohistochemistry (IHC) in liver tissues of patients with HCC, LC or normal liver.

## 2. Materials and Methods

### 2.1. Ethics

In the UK, the study was approved by both the Coventry and Warwickshire and the Northeast York Research National Health Service Ethics Committees (Reference numbers 09/H1211/38 and 19/NE/0213). To access stored tissue samples for the purpose of immunohistochemistry, we were granted an ethical approval from the Arden Tissue Bank, UK (Reference No. ATB19-013). In Germany, the study was approved by the Ethics Committee of the Medical School Hannover (Reference number: 901). The study conformed to the World Medical Association Declaration of Helsinki, with all study participants providing written informed consent.

### 2.2. Study Design

This was a prospective cross-sectional study that included a discovery phase and a validation phase. Participants in the study were recruited between 1 January 2013 and 30 June 2019 at both University Hospital Coventry and Warwickshire, UK and Hannover Medical School, Germany. A follow-up period to note death outcomes was closed on 15 November 2020. In the discovery phase, we prospectively recruited 18 HCC cases and 51 non-HCC cases, while, in the validation phase, we prospectively recruited 39 HCC and 87 non-HCC cases. Diagnosis of these patients was established by a combination of liver ultrasound, laboratory markers, Fibrosis 4 index (FIB-4), CT/MRI scans, and histology. The HCC diagnosis was in line with international diagnostic criteria used in Europe [3]. HCC patients were recruited before receiving anti-cancer treatment. Clinical characteristics of recruited patients are demonstrated in Table 1. Graphical abstract and schematic flow chart showing the different phases of the study are shown in Supplementary Figures S1 and S2.

### 2.3. Sample Preparation

Five mL of urine was collected from all study participants in standard universal specimen containers (Newport, UK) and was frozen to −80 °C, after collection, for subsequent batch analysis. For proteomic analysis, the urine samples were prepared as previously described [18]. In brief, a 0.7 mL aliquot was thawed immediately before use and diluted with 0.7 mL 2 M urea, 10 mM NH_4_OH containing 0.02% Sodium Dodecyl Sulphate. To remove proteins of higher molecular mass (e.g., albumin and immunoglobulin G), the sample was filtered using a Centrisart ultracentrifugation filter device (20 kDa molecular weight cut-off; Sartorius, Göttingen, Germany) at 3000 rcf until 1.1 mL filtrate was obtained. Subsequently, the filtrate was loaded onto a PD-10 desalting column (GE Healthcare, München, Germany), and equilibrated in 0.01% NH_4_OH in HPLC-grade H_2_O (Roth, Karlsruhe, Germany) in order to decrease matrix effects by removing urea, electrolytes, and salts, and also to enrich polypeptides. Finally, all samples were lyophilized, stored at 4 °C, and resuspended in HPLC-grade H_2_O shortly before CE-MS analysis.

### 2.4. CE-MS Analysis

CE-MS analysis was performed using a P/ACE MDQ capillary electrophoresis system (Beckman Coulter, Fullerton, CA, USA) online coupled to a Micro Time-of-Flight MS (Bruker Daltonic, Bremen, Germany) as described [19]. For CE-MS coupling, the ESI sprayer (Agilent Technologies, Palo Alto, CA, USA) was grounded, ion spray interface potential set between −4.0 and −4.5 kV and MS acquisition methods automatically controlled by the CE via contact-close-relays. Spectra were accumulated every 3 s over a range of m/z 350 to 3000. Details on accuracy, precision, selectivity, sensitivity, stability, and reproducibility of the CE-MS method have been established [20].

### 2.5. CE-MS Data Processing

Mass spectral ion peaks, representing identical peptides at different charge states, were deconvoluted into single masses using MosaiquesVisu software [21]. For noise filtering, signals with z > 1 observed in a minimum of 3 consecutive spectra with a signal-to-noise ratio of at least 4 were considered. MosaiquesVisu employs a probabilistic clustering algorithm and uses both isotopic distribution (for z ≤ 6) and conjugated masses for charge-state determination of peptides/proteins. The resulting peak list characterizes each peptide by its mass and its migration time. Time-of-flight-MS data were calibrated utilizing 150 reference mass data points and 452 reference migration time data points by applying global and local linear regression, respectively. Ion signal intensity (amplitude) varied between samples, mostly due to different amounts of salt and peptides in the sample and were therefore normalized. Reference signals of 29 highly abundant peptides were used as “internal standard” peptides for calibration using local linear regression [22]. This procedure was shown to be an easy and reliable method to address both analytical and dilution variances in a single calibration step. The obtained peak list characterizes each peptide by its calibrated molecular mass [Da], calibrated CE migration time [min], and normalized signal intensity. All detected peptides were deposited, matched, and annotated in a Microsoft SQL database allowing further statistical analysis. The raw data files are uploaded to the open access database Zenodo; https://zenodo.org/ and are linked to the DOI: 10.5281/zenodo.5138595 last accessed on 21 July 2021.

### 2.6. Support Vector Machine Model Generation and Classification

For the integration of a set of peptides to a support vector machine (SVM) classification model, the MosaCluster v.1.7.5 software was applied (Biomosaiques Software GmbH, Hannover, Germany). MosaCluster constructs a high-dimensional parameter space based on the amplitudes of the selected peptides and defines a separation hyperplane between two groups defined as case or control during the supervised learning phase. After establishment, such an SVM peptide marker model can be used for diagnosis by assigning to each patient’s CE-MS profile a membership value according to the level of similarity to either the case or control group used for training. To compensate for imbalanced data, MosaCluster includes a class-weighting function based on the ratio of the two classes, which is used for assigning higher misclassification penalties to the larger group.

### 2.7. Peptide Sequencing

Peptide sequencing was carried out both on a Dionex Ultimate 3000 RSLS nanoflow system (Dionex, Camberley, UK) and a Beckman CE/Orbitrap Q Exactive plus combination (Thermo Scientific, Waltham, MA, USA) [23]. Spectra files were analyzed with Proteome Discoverer 2.4 (Thermo Scientific) allowing a precursor mass tolerance of 5 ppm and a fragment mass tolerance of 0.05 Da. This was followed by a search using the SEQUEST search engine against the UniProt human non-redundant database (https://www.uniprot.org/, last accessed on 9 March 2021) without any protease specificity or fixed modification. Oxidation of methionine and proline were considered as variable modifications. Only sequences with high confidence (Xcorr ≥ 1.9) and without unmodified cysteine (due to the application of non-reducing conditions) were accepted [24]. A strong correlation between peptide charge at the CE operating pH of 2 deduced from the number of basic amino acids in the annotated peptide sequence and the migration time was used as another criterion to prevent false sequence assignments [25].

### 2.8. In Silico Protease Prediction

In silico protease assessment was performed using Proteasix (www.proteasix.org, last accessed on 18 March 2021), the web-based tool for investigation of proteolytic events involved in naturally occurring peptide generation [17]. Observed specific proteases responsible for cleavage of N- or C- terminus of a peptide were retrieved from CutDB proteolytic event database available at www.cutdb.burnham.org (last accessed on 18 March 2021) [26]. Protease activity was assessed in the patient’s CE-MS peptide profiles to gain fold-changes between HCC cases (*n* = 18) versus the controls with other cirrhotic and non-cirrhotic liver diseases (*n* = 51) based on the average of associated peptide intensities. This method is described in detail by Voigtländer et al. [15].

### 2.9. Immunohistochemistry

To further evaluate the presence of kallikrein-6 (KLK6) and meprin A subunit α (MEP1A), as the two proteases with the highest activity score according to Proteasix, the Arden tissue bank provided us with liver tissue sections. We extracted 14 cases (5 with HCC, 4 with benign liver disease including cirrhosis and 5 cases with normal liver tissue without disease). For both KLK6 and MEP1A detection, we used commercial polyclonal goat IgG antibodies known to react to human tissue (R&D Systems, Abingdon, UK). We first optimized the antibodies; the human protein atlas was queried, to investigate the current guidance on antibody dilutions and anticipated staining patterns for both KLK6 and MEP1A. Following optimization on test cases, we used a KLK6-specific primary antibody at a dilution of 1:200 and MEP1A-specific primary antibody at a dilution of 1:1400 for all immunohistochemistry. Each tissue section was reviewed by a Gastrointestinal Pathologist to ensure that adequate tissues were present prior to staining. For immunohistochemistry assessment of the detected protease, the Allred Scoring system for stain intensity was used; 0 for Negative (no staining of any nuclei at high magnification), 1 for weak (only visible at high magnification), 2 for Moderate (readily visible at low magnification), and 3 for strong (strikingly positive at low magnification) [27]. Detailed steps for immunohistochemistry are described in the supplementary material.

### 2.10. Statistics

*p*-values for group-specific differences in peptide distributions were calculated based on natural logarithmic transformed ion signal intensities and the Wilcoxon rank sum test using the statistical programming language R. Statistical adjustment of *p*-values due to multiple testing was performed by the Benjamini and Hochberg method [28]. All other statistical analyses were carried out using the statistical software MedCalc version 12.7.5.0 (MedCalc Software; Mariakerke, Belgium). Receiver operating characteristics (ROC) analysis was used to determine estimates of sensitivity and specificity for classification also including exact binomial calculations for confidence intervals. ROC analysis and the determination of AUC values thereof were used as these are accepted descriptors to determine diagnostic test accuracy. A major characteristic of ROC analysis is that it describes the classifier’s performance over the entire range of criterion values and therefore provides the advantage to be independent of any particular threshold. The relationship between proteomic classification to demographic variables was performed by binomial logistic regression analysis. Overall survival was analyzed by Kaplan–Meier methodology and a log-rank test to compare patients with a positive test result versus those with a negative test result by the HCC proteomic test.

## 3. Results

### 3.1. Identification of Urinary Peptides as HCC Progression Markers by CE-MS Analysis

The urine samples of the HCC study cohort were analysed by CE-MS resulting in a list of 7259 peptides in the molecular mass range between 800 and 20,000 Dalton and with a frequency of occurrence in at least 20% of samples. A threshold of 20% was chosen to have on the one side a sufficient high parameter space and on the other side can still handle the zero-inflated data matrix of peptide signal amplitudes in the CE-MS peptide profiles. The latter criterion is of particular relevance for differential analysis of single peptide distributions.

In order to identify urinary peptides with differential regulation between HCC case and normal or liver fibrotic control groups, we followed a two-step selection approach. Firstly, we performed a group-wise comparison of 18 urine samples from HCC patients and 51 non-HCC controls (25 LC, 8 NASH w/o LC, 9 NAFLD, 9 center-matched healthy individuals) by a parametric Wilcoxon rank sum test. This resulted in the identification of 123 peptides with a *p*-value below 0.05 after false discovery rate (FDR) adjustment by the method of Benjamini and Hochberg [28]. Using this extended set of markers, we subsequently performed a search for those peptide markers that showed a gradual increase or decrease in their CE-MS-detected amplitude signals from normal or non-cirrhotic liver disease including NAFLD and NASH without cirrhosis over LC of different aetiology to HCC (for details on patient characteristics see Table 1). This selection procedure resulted in a list of 31 out of the set of 123 peptides with significant Spearman Rho correlation coefficients after FDR adjustment either above 0.3 or below −0.3 defining a source of HCC progression markers. The 31 peptides with significant association to HCC including all their statistical characteristics are presented in Table 2.

### 3.2. Development of the 31 HCC Progression Markers to a Multivariate Classification Model

The 31 selected peptides were combined to a support vector machine (SVM)-based peptide model. This SVM model, named HCC-31, was trained during the supervised learning phase using the 18 HCC and 51 non-HCC control patients of the discovery study cohort to differentiate between HCC and non-HCC specific peptide marker patterns. Concerning SVM characteristics, HCC-31 is based on a radial basis function (RBF) kernel of C-SVC type with C = 2.2691, g = 0.0764 and eps = 0.001 as fixed kernel parameter settings. Selection of an RBF kernel of C-SVC was found to be the best option for data matrices with frequent occurrence of zero intensity values as represented by CE-MS peptide profiles [29]. After optimization of the SVM parameters and total cross-validation on the original training data, the peptide marker pattern resulted in an AUC of 0.92 (95% confidence interval (CI): 0.87 to 0.96, *p* < 0.0001) in receiver operating characteristics analysis (ROC). The optimal threshold for an HCC positive test result was determined based on the Youden index to be −0.25 resulting in a sensitivity of 86.8% (95% CI: 74.7–94.5) and a specificity of 89.0% (95% CI: 81.2–94.4).

**Figure 1:**
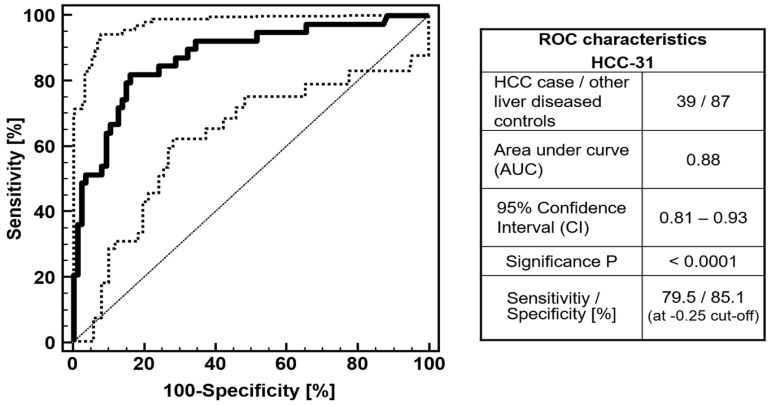
ROC characteristics of the proteomic HCC classification model HCC-31 on the validation set of 39 HCC cases and 87 non-HCC liver diseased controls.

In order to determine the model’s accuracy without overfitting bias, the HCC-31 model was subsequently tested on an independent cross-sectional cohort of patients of whom 39 had a clinical diagnosis for HCC and 87 for other liver diseases. As presented in Figure 1, independent validation of the HCC-31 model resulted in an AUC of 0.88 (95% CI: 0.81–0.93, *p* < 0.0001), and 79.5% sensitivity and 85.1% specificity at the predetermined threshold at −0.25. When classification by the HCC-31 model was adjusted for age and gender of the patients in the validation cohort, the AUC in ROC analysis was significantly increased from 0.88 to 0.94 (*p* = 0.008). Based on the age- and gender-matched HCC-31 model, only two out of the 39 HCC cases were missed, as they were classified as controls (false negatives).

**Figure 2:**
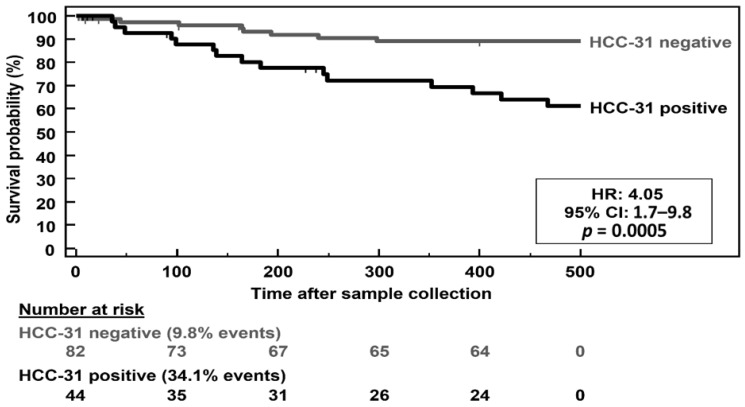
Overall survival of patients with a positive or negative HCC-31 test result during a follow-up of 500 days starting from the date of urine sample collections. Patients lost to follow-up were censored.

Subsequently, we investigated the prognostic value of a positive HCC-31 test, by investigating whether the classification result is a significant predictor of overall mortality during a follow-up period of 500 days starting from the date of sample collection. As revealed by the Kaplan–Meier survival curves in Figure 2, patients with a positive HCC-31 test had a 4.1-fold increased risk of death (95% CI: 1.7–9.8, *p* = 0.0005) compared to patients with a negative test during the 500-days follow-up.

### 3.3. CE-MS and Peptide Sequence Characteristics of the Peptide Marker Candidates

For the 31 peptides that were identified as differentially excreted in the urine between HCC cases and disease matched controls, amino acid sequences were assigned based on mapping of the CE-MS characteristics (CE migration time and MS-detected molecular mass) to the urinary peptide sequence database [30]. The CE-MS characteristics for the 31 peptides together and the sequence information for all sequence identified peptides (*n* = 27) are presented in Table 3.

Following the hypothesis that peptides emerge from proteolytical processing of proteins and that peptides serve as substrates of disease-specific changes to the proteolytic environment, in silico mapping was performed on the 27 sequence identified HCC peptide marker of the HCC-31 model. In total, 18 protease candidates were found to be associated with the sequence motifs at the N- and C-terminal ends of the 27 peptides. Out of these, seven showed significant differences in the ion signal intensities of their mapped peptide substrates between the HCC case and other liver disease control groups after adjustment for multiple testing. As presented in Table 4, kallikrein-6 (KLK6), the matrix metallopeptidase (MMP) 3 and 13 and the cathepsins (CTS) D and E were predicted to be significantly increased, whereas meprin A subunit α (MEP1A) and CTSB were found to be decreased in their activities in HCC compared to non-HCC liver diseases (*p* < 0.05 in the Mann–Whitney U test).

### 3.4. Differential Expression of KLK6 and MEP1A in HCC, Cirrhosis and Normal Liver Tissue

**Figure 3:**
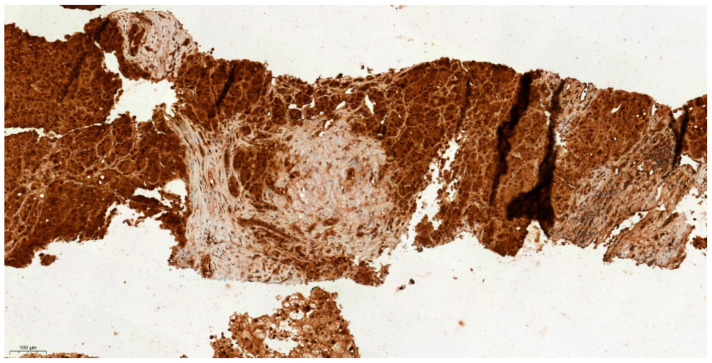
IHC characteristics of kallikrein-6 (KLK6) in HCC at low power magnification (×10). The brown chromogen indicated protein expression of KLK6 in the nucleus and cytoplasm of the hepatic and stromal cells. Slide shows infiltrating nests and cords of atypical hepatocytes in the stroma with mildly pleomorphic nuclei in keeping with moderately differentiated hepatocellular carcinoma, demonstrating high intensity of nuclear and cytoplasmic staining for KLK 6 (Allred score 3).

**Figure 4:**
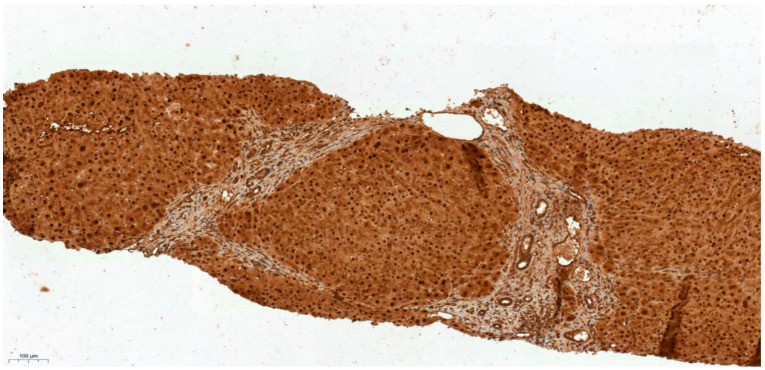
IHC characteristics of kallikrein-6 (KLK6) in liver cirrhosis at low power magnification (×10). The brown chromogen indicated protein expression of KLK6 in the nucleus and cytoplasm of the hepatic and stromal cells. The slide shows a section of a cirrhotic liver showing well defined nodules of regenerating hepatocytes with expanded fibrotic portal tracts without marked nuclear atypia—demonstrating moderate intensity of nuclear and cytoplasmic staining for KLK 6 (Allred score 2).

**Figure 5:**
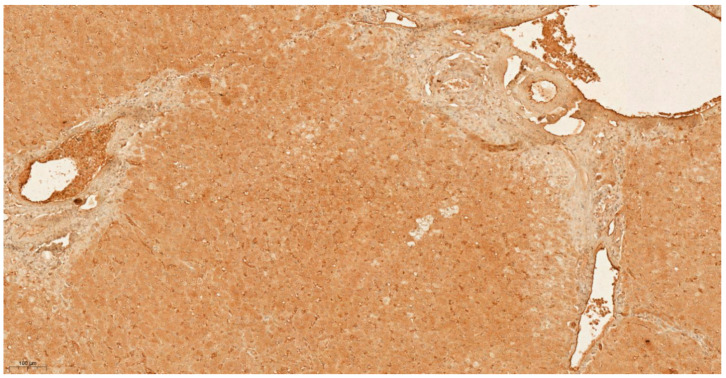
IHC characteristics of kallikrein-6 (KLK6) in normal liver at low power magnification (×10). The brown chromogen indicated protein expression of KLK6 in the nucleus and cytoplasm of the hepatic and stromal cells. The slide shows a section of a normal liver containing normal appearing portal tracts and hepatocytes—demonstrating mild intensity of nuclear and cytoplasmic staining for KLK6 (Allred score 1).

**Figure 6:**
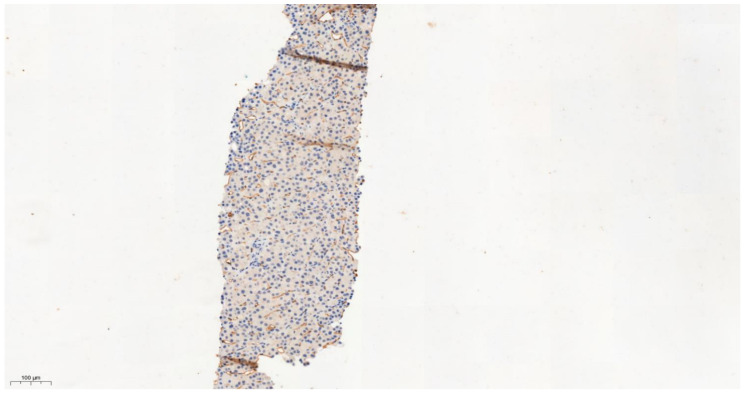
IHC characteristics of meprin A subunit a (MEP1A) in HCC at low power magnification (×10). The brown chromogen should indicate protein expression of MEP1A in the nucleus and cytoplasm of the hepatic and stromal cells. The slide section shows compact sheets and nests with thickened hepatocyte plates with mild cytological atypia in keeping with well differentiated hepatocellular carcinoma; staining for MEP1A here was negative (Allred score 0).

**Figure 7:**
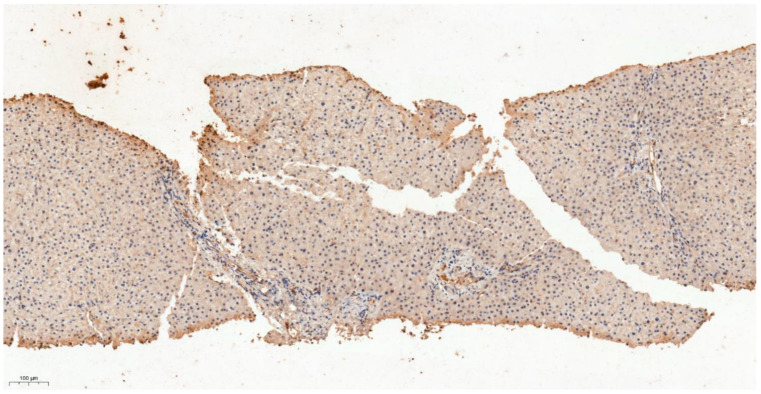
IHC characteristics of meprin A subunit a (MEP1A) in liver cirrhosis at low power magnification (×10). The brown chromogen should indicate protein expression of MEP1A in the nucleus and cytoplasm of the hepatic and stromal cells. The slide section of cirrhotic liver shows vague nodule formation with bridging fibrosis; staining for MEP1A here was negative (Allred score 0).

**Figure 8:**
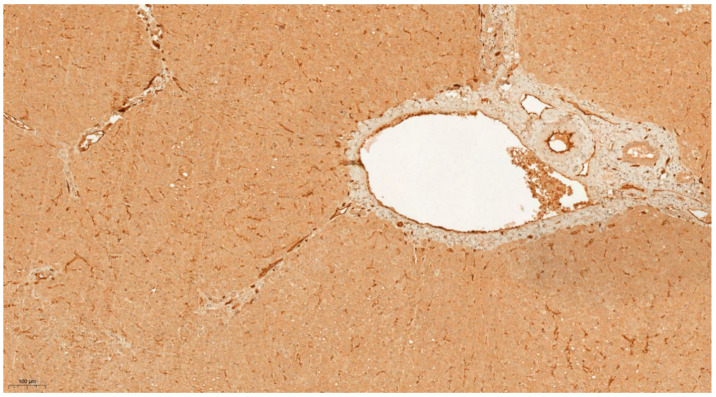
IHC characteristics of meprin A subunit a (MEP1A) in normal liver at low power magnification (×10). The brown chromogen indicated protein expression of MEP1A in the cytoplasm of the hepatic and stromal cells. The slide section of a normal liver contains normal appearing portal tract and hepatocytes—demonstrating mild intensity of nuclear and cytoplasmic staining for MEP1A (Allred score 1).

The proteases KLK6 and MEP1A were selected for immunohistochemical (IHC) staining of liver biopsy sections since they showed the highest difference in activity between HCC cases and controls in Table 4. For the investigation of KLK6 and MEP1A tissue expression, liver biopsy sections from five HCC, four benign liver disease including cirrhosis and five cases with normal liver tissue without disease were selected from the Arden tissue bank. As shown in Figure 3, Figure 4 and Figure 5, incremental gradient staining ranging from mild staining in normal liver tissue, to moderate staining in liver cirrhosis and then to strong diffuse staining in HCC was observed for KLK6. For MEP1A, there was an absence of staining in cirrhosis and HCC, whereas it was mildly present in normal liver tissue (Figure 6, Figure 7 and Figure 8). The IHC suggests that KLK6 increase with cirrhosis and HCC while MEP1A decrease in cirrhosis and HCC. For gradient staining, we used the Allred score [27], and this is demonstrated in all tested histopathological sections in Table 5.

## 4. Discussion

There are no accurate diagnostic biomarkers for HCC or population-based screening. Additionally, surveillance strategies for HCC are ineffective, relying on liver ultrasound scans (USS) for the detection of nodules in LC patients, which is dependent on the quality of training of the USS operator. The role of a fetoprotein (AFP) in HCC surveillance is also questionable due its poor sensitivity and is no longer recommended for routine use. HCC diagnosis relies mainly on the ability of advanced, high-resolution imaging techniques for the detection of liver lesion early arterial enhancement followed by early washout. These scans are not easily accessible and can be less accurate in detecting lesions <1–2 cm. The current modalities used are contrast-enhanced triphasic computed tomography (CT) and/or contrast-enhanced magnetic resonance imaging (MRI). If the scans are inconclusive, the diagnosis is then confirmed with a cytological or histopathological evaluation of the liver lesion from tissue biopsy. Treatment and prognostication of patients with HCC consider the size and number of tumour nodules and their relation to the portal vein, and the degree of liver impairment [2,3,4]. Given these factors, there is a need for non-invasive methods to identify HCC.

In this respect, the present study was focused on the identification of HCC-specific peptides in urine to first test their diagnostic utility by integration into a peptide marker model as previously performed for cholangiocarcinoma [15] and pancreatic cancer [31] and second to trace back systemic alterations of HCC progression to pathophysiological processes at the tumor site.

The HCC-31 classifier adds to the current modalities for non- or minimal-invasive HCC diagnosis. To put this in a clinical perspective, the HCC-31 performed better in comparison to AFP. HCC-31 showed sensitivity of 79.5% while the quoted literature showed that AFP usually has low sensitivity for HCC detection between 40–65% [32]. Therefore, potential use of HCC-31 is promising if further validated as substitute to AFP in aiding HCC diagnosis or as a prognostic marker.

HCC-31 utilizes a molecular pattern of 31 peptides, which are surrogate markers for differential proteolytic activity at the HCC tumor site in comparison to other cirrhotic and non-cirrhotic liver diseases. Validation of HCC-31 on an independent cross-sectional cohort of 39 HCC and 87 highly heterogeneous non-HCC liver disease patients from two clinical populations, one in England and the other in Germany, resulted in an accuracy of 83.3% of the pure classification model and 91.3% when the model was adjusted for gender and age. Moreover, HCC-31 positivity was associated with a 4-fold increased risk of death during a 500-day observational period providing further evidence for its clinical applicability.

The 31-HCC model consists of peptides derived from different protein sources including cell-derived and structural proteins. As revealed by a literature review, several of the HCC-31 peptide markers were also identified in other human body fluids, like serum, plasma, cerebrospinal fluid, or as HLA-associated immunopeptides in tissue and are therefore proven not to be restricted to urine (for details, see Table 6) [33,34,35,36,37,38,39].

Some of the proteins from which the peptide markers are derived are already described in the context of HCC, such as CDH1/E-cadherin [40] and AHNAK [41], but most others are not. Peptides as disease markers add another level of complexity since their expression differences might not be caused by differential regulation of their parent protein but changes in the activity of the proteases leading to their generation. Therefore, the common features qualifying the 31 peptides as HCC markers are most likely their cleavage by proteases with changes in activity during the course of HCC and the same route of clearance by incorporation into exosomes, release into the circulation and final excretion into the urine. Most of the peptides included in HCC-31 are fragments of collagen chains, which are also identified as source of peptide markers for other diseases by our group [42,43].

Collagen chains are the main components of the extracellular matrix, and their fragments are predominant in the low molecular weight fraction of the urinary proteome [44]. Various proteases are able to cleave collagen chains, most prominent are matrix metalloproteinases and cathepsins [45]. In this respect, we were able to detect more than 600 different partially overlapping peptides derived from collagen α-1(I) chain in urine (unpublished data). As already described in other studies by our group [46,47], the composition of collagen peptides in urine is strongly associated with changes in specific protease activities at the site of disease, particularly in progressing tumours and surrounding microenvironment.

Carcinogenesis exact mechanisms are yet to be identified. However, cancer cells’ metabolism involves extracellular proteolytic degradation. This mainly plays a role in cell migration, tumour growth and distant spreading in the body [48]. Therefore, investigations at the protein level (proteomics) are advantageous particularly in the case of in-depth characterization of cancer progression and invasiveness. CE-MS has demonstrated in this context a good diagnostic potential of urinary peptide biomarkers even for non-renal diseases with exosomes as the potential trans-renal carriers. These biomarkers have been identified in the context of a single type of cancer (e.g., bladder, prostate, pancreatic, renal cell carcinoma and cholangiocarcinoma) [31,47,49,50]. Our results here have demonstrated that proteolytically processed peptides in the urine can be used in diagnosis and prognosis of HCC, and this is actually a promising non-invasive tool for precision medicine in the future. We have also demonstrated that these urinary peptides are related to proteolytic activities at the tumour site. We chose to demonstrate the predicted proteases in various stages of liver tissue ranging from normal to cirrhosis and HCC to identify firstly if these proteases are present and if their staining differs between normal and disease groups, namely LC and HCC. KLK6 and MEP1A were shortlisted as per the lowest *p*-value. The gradient staining confirmed the predicted activity, showing that KLK6 increases with cirrhosis and HCC, and MEP1A decreases in cirrhosis and HCC.

KLK6 is a protease that belongs to the kallikrein family of fifteen members located on chromosome 19. KLK6 was shown to be involved in many cancers’ formation and progression [51,52,53,54]. In the liver, KLK6 was shown to catalyse ubiquitin, an important cellular regulatory protein involved in protein synthesis. KLK6 also was shown to induce de novo cirrhosis and was increased in HCC tissues [55]. Additionally, a study designed to check the activity of KLK6 on ECM peptides in HCC revealed that KLK6 has an upregulated activity [56].

MEP1A is a metalloproteinase that belongs to the metzincin family with the main function in intracellular transport of proteins [57]. MEP1A has been implicated in kidney, colorectal and pancreatic cancers [31,58,59]. In HCC, MEP1A was shown to promote cell proliferation, migration and invasion [60,61]. In the present study, we have shown that MEP1A related peptides in the urine are present in HCC at decreased levels than in our cirrhosis and non-cirrhosis control group. However, both the staining in cirrhosis and HCC tissues were negative but present in normal livers. This was also noted by OuYang et al. [61] on HCC tissues, where immunohistochemical MEP1A expression levels in the tumour cell cytoplasm varied widely among different HCC specimens. However, the same group showed that MEP1A was found to be elevated following analysis of the HCC tissues using quantitative real-time polymerase chain reaction compared with matched adjacent nonneoplastic tissues and non-malignant liver disease tissues. Differential regulation in this respect might occur on the protein level, e.g., by secretion of soluble MEP1A, rather than forming a membrane-bound complex within the cell or on the cell surface [62]. In addition, the presence of MEP1A in HCC tissues also demonstrated poor prognosis [61].

The predicted seven proteases in this study could also be potential sites for anti-protease treatment in HCC. An example was demonstrated in a study by Tran et al. [63]. They showed that injection of metalloproteinases (MMPs) inhibitors to HCC cell lines resulted in delaying HCC growth without treatment related toxicity. MMP inhibitors also lead to inhibition of angiogenesis and tumour necrosis. Furthermore, anti-cathepsins were found to promote cell death in a study completed on HepG2 cell lines [64]. These anti-proteases could be used through an immunotherapy approach in combination with conventional chemotherapy and/or nanoparticle based intervention.

CE-MS technology has identified an important sequence of urinary peptides related to proteolytic activity in HCC. The technology paves the way for future work on these peptides to develop a noninvasive test that could be applied early for purpose of screening, surveillance and/or diagnosis. The study was limited by the relatively small number of patients, small number of human liver tissue samples and its exploratory nature; nonetheless, it was multicentre and validated across two populations. In addition, the presentation of the predicted proteases was verified at the tissue level demonstrating that these urinary peptides are related to the HCC disease formation in the liver.

## 5. Conclusions

Urinary CE-MS analysis identified proteases specific to HCC. In addition, the specific HCC peptide model showed good diagnostic performance and prognostic ability in relation to outcomes.

## Figures and Tables

**Table 1:** Clinical and demographic data of HCC case and non-HCC control patients included in the discovery and validation phase of the study. Parameters, demonstrating significant differences between the HCC and non-HCC groups, were further investigated using one-way ANOVA on subgroups of patients with NAFLD (*n* = 27), NASH without LC (*n* = 14), LC (*n* = 72) and HCC (*n* = 57). The respective Box-and-Whisker distribution plots are presented in Supplementary Figure S3.

Study Phase	Discovery				Validation		
Patient Group	HCC	Non-HCC	*p* *		HCC	Non-HCC	*p* *
Patients/samples, *n*	18/18	51/51	n.a.		39/39	87/87	n.a.
Age, years, mean/range	58/28–76	52/18–82	0.08		67/38–87	56/20–85	0.0001
Female/male, *n*	3/15	20/31	0.14		9/30	35/52	0.07
No. detected peptides, mean/range	1753/623–2965	2329/920–4488	0.02		2872/1073–4617	2461/811–4057	0.008
Liver cirrhosis, *n* (%)	18 (100)	25 (49)	<0.0001		28 (72)	47 (54)	0.08
Diabetes mellitus, *n* (%)	3 (17)	13 (25)	0.53		16 (41)	23 (26)	0.14
Body mass index, mean/range	26.8/18.3–32.1	27.8/20.3–41.7	0.76		27.7/20.2–41.0	27.5/16.4–46.6	0.52
Platelet count, ×10^9^/L, mean/range	99/26–310	162/25–391	0.02		195/28–595	234/44–961	0.04
a-Fetoprotein (AFP), µg/L, mean/range	1821/3–22,826	4/1–50	<0.0001		6669/1–107,202	39/1–1493	<0.0001
Alkaline phosphatase, U/L, mean/range	151/60–380	125/45–797	0.008		291/83–1781	196/37–693	0.02
Aspartate aminotransferase (AST), U/L, mean/range	74/24–284	49/13–120	0.03		112/26–457	69/13–1606	<0.0001
Alanine aminotransferase (ALT), U/L, mean/range	61/14–288	53/10–304	0.84		69/12–242	72/7–1038	0.37
AST:ALT ratio, mean/range	1.48/0.89–3.41	1.17/0.20–3.20	0.05		2.11/0.55–7.46	1.13/0.18–3.06	0.0004
Approx. Ishak Fibrosis Score as per FIB-4 index, *n*, 0–1/2–3/4–6	0/1/17	14/12/17	0.0004		2/9/28	36/28/23	<0.0001
Albumin, g/L, mean/range	32/19–48	40/26–51	0.0003		34/16–48	38/15–70	0.01
Bilirubin, µmol/L, mean/range	27/9–86	26/3–163	0.05		43/6–254	34/3–390	0.03
ALBI stage, *n*, 1/2/3	2/11/5	26/15/4	0.003		11/20/8	47/22/18	0.009
**Liver Disease Etiology, *n***							
Primary HCC	0	0			1	0	
Alcoholic cirrhosis (C2)	7	4			7	6	
Virus-related cirrhosis (HBV/HCV/HDV)	3/4/0	0/4/1			3/4/0	2/2/0	
Cryptogenic/Biliary cirrhosis	0/0	3/1			1/0	2/0	
Hereditary (Mucoviscidosis/Hemochromatosis/AATD)	1/1/0	0/0/0			1/0/1	0/0/0	
Cholestasis (PBC/PSC/SSC/PFIC)	2/0/0/0	2/2/1/1			2/5/0/0	2/14/1/0	
Autoimmune hepatitis (AIH)	0	2			0	0	
NAFLD/NASH/NASH-LC	0/0/0	9/8/4			0/2/12	18/6/15	
GI cancer (CCA/PCA) with LC	0/0	0/0			0/0	7/2	
Other benign liver diseases (CHP/CDL)	0/0	0/0			0/0	7/3	
No liver disease	0	9			0	0	

* Mann–Whitney U test for continuous data and significance level by Fisher’s exact or chi-squared test for categorical data. Abbreviations: AATD, a-1 antitrypsin deficiency; CCA, cholangiocarcinoma; CDL, choledocholithiasis; CHP, chronic pancreatitis; HCC, hepatocellular carcinoma; LC, liver cirrhosis; NAFLD, non-alcoholic fatty liver disease; NASH, nonalcoholic steatohepatitis; PBC, primary biliary cholangitis; PCA, pancreatic cancer; PFIC; progressive familial intrahepatic cholestasis; PSC, primary sclerosing cholangitis; SSC, secondary sclerosing cholangitis.

**Table 2:** Statistical characteristics and peptide marker distributions in the HCC case and non-HCC liver disease control discovery groups of the 31 urinary peptides included in the HCC peptide marker model.

Peptide ID ^†^	Group-Wise Comparison ^§^ of Peptide Distributions HCC (*n* = 18) vs. Non-HCC Liver Disease (*n* = 51)		Rank Correlation of Peptide Amplitudes to a Grading Score 0 = Non-LC ^#^, 1 = LC & 2 = HCC		Peptide Distribution in the Discovery Patient Groups					
					Non-LC ^#^ (*n* = 26)		LC (*n* = 25)		HCC (*n* = 18)	
	*p*-Value ^‡^ for Group Differences	AUC for Group Differences	Spearman Rho Coef.	*p*-Value ^‡^ for Rank Differences	Mean Amp (SD)	Freq.	Mean Amp (SD)	Freq.	Mean Amp (SD)	Freq.
54	8.34 × 10^−03^	0.70	0.353	1.43 × 10^−02^	2 (10)	4	7 (28)	12	53 (85)	56
1059	4.57 × 10^−02^	0.63	0.303	4.06 × 10^−02^	4 (18)	8	3 (13)	12	30 (55)	33
1160	4.23 × 10^−02^	0.63	0.431	1.83 × 10^−03^	1 (4)	8	34 (46)	48	61 (81)	67
1778	1.50 × 10^−02^	0.65	0.407	3.66 × 10^−03^	5 (26)	4	18 (34)	32	29 (40)	50
2314	2.92 × 10^−02^	0.65	0.385	6.52 × 10^−03^	1911 (3077)	42	2696 (2346)	80	3017 (2222)	94
3559	1.30 × 10^−02^	0.63	0.396	4.97 × 10^−03^	47 (88)	31	184 (223)	60	201 (224)	67
3662	8.27 × 10^−04^	0.76	0.489	3.08 × 10^−04^	76 (91)	62	161 (188)	76	256 (177)	89
4564	6.81 × 10^−03^	0.72	0.479	4.18 × 10^−04^	270 (130)	92	639 (579)	96	591 (449)	94
4610	2.50 × 10^−03^	0.65	0.398	4.74 × 10^−03^	0 (0)	0	64 (157)	16	166 (272)	39
4829	1.54 × 10^−02^	0.66	0.409	3.49 × 10^−03^	59 (142)	27	277 (358)	76	245 (286)	67
5284	5.61 × 10^−03^	0.70	0.362	1.15 × 10^−02^	63 (102)	42	81 (137)	44	172 (174)	67
6601	3.57 × 10^−06^	0.72	0.470	5.54 × 10^−04^	0 (0)	0	1 (4)	4	199 (286)	44
6607	8.67 × 10^−04^	0.65	0.380	7.39 × 10^−03^	0 (0)	0	19 (83)	8	259 (737)	33
8720	6.37 × 10^−04^	0.75	−0.587	8.05 × 10^−06^	658 (664)	73	231 (641)	48	31 (98)	11
9080	3.34 × 10^−02^	0.65	0.391	5.69 × 10^−03^	813 (2125)	15	893 (1236)	48	1618 (1815)	61
9510	5.03 × 10^−03^	0.72	0.370	9.56 × 10^−03^	2227 (4587)	31	2847 (4477)	68	4563 (3380)	83
9728	1.90 × 10^−03^	0.73	0.511	1.51 × 10^−04^	345 (745)	23	902 (1038)	56	1359 (916)	89
10177	3.24 × 10^−03^	0.71	0.358	1.29 × 10^−02^	162 (288)	38	170 (224)	48	476 (376)	89
11725	6.63 × 10^−04^	0.65	0.385	6.64 × 10^−03^	0 (0)	0	4 (12)	8	28 (50)	33
12459	8.13 × 10^−06^	0.86	−0.526	8.68 × 10^−05^	373 (152)	96	335 (236)	92	119 (124)	61
13134	7.85 × 10^−04^	0.73	0.311	3.45 × 10^−02^	32 (73)	27	170 (375)	24	364 (411)	67
13176	1.20 × 10^−03^	0.73	0.410	3.47 × 10^−03^	27 (54)	27	103 (190)	40	171 (155)	72
14389	5.32 × 10^−04^	0.74	−0.400	4.53 × 10^−03^	53 (64)	54	20 (37)	40	0 (0)	0
14925	7.69 × 10^−04^	0.73	−0.567	1.82 × 10^−05^	689 (1120)	58	237 (627)	32	0 (0)	0
15342	1.38 × 10^−04^	0.80	−0.604	3.66 × 10^−06^	766 (308)	100	420 (323)	76	224 (219)	78
17066	1.33 × 10^−04^	0.66	0.397	4.89 × 10^−03^	0 (3)	4	8 (53)	4	257 (693)	33
17805	8.75 × 10^−04^	0.75	−0.514	1.37 × 10^−04^	394 (478)	69	235 (349)	52	16 (36)	22
19681	5.57 × 10^−04^	0.76	−0.630	1.14 × 10^−06^	127 (129)	85	34 (53)	40	9 (21)	17
20237	1.82 × 10^−04^	0.79	0.483	3.69 × 10^−04^	292 (480)	35	316 (635)	56	940 (1064)	94
24328	2.07 × 10^−05^	0.84	−0.636	8.84 × 10^−07^	5557 (3207)	100	2341 (2674)	100	890 (1187)	72
29919	1.20 × 10^−02^	0.69	0.465	6.63 × 10^−04^	199 (505)	23	525 (661)	60	857 (1223)	78

^†^ Peptide identification number. ^§^ acc. non-paramteric Wilcoxon signed rank sum test. ^#^ non-LC group with normal controls (*n* = 9), NAFLD (*n* = 9) and NASH w/o LC (*n* = 8). ^‡^ Correction for multiple testing by the method of Benjamini and Hochberg. Abbreviations: AUC, area under curve; Freq, frequency of occurrence (%); HCC, hepatocellular carcinoma; LC, liver cirrhosis; Mean Amp, mean signal amplitude (ion counts); *n*, number of patients; NAFLD, non-alcoholic fatty liver disease; NASH, nonalcoholic steatohepatitis; SD, standard deviation.

**Table 3:** Characterization of the 31 urinary HCC peptide markers by amino acid sequencing and in silico protease prediction analysis together with their experimental spectrometry mass, retention time in capillary electrophoresis, and location in the proteins linear sequence.

Peptide ID ^†^	Exp. Mass [Da]	CE Time [min]	Protein	AA	Proteases for N-Terminal Cleavage	Peptide Sequence (Black) with Flanking Regions (Grey) ^‡^	Proteases for C-Terminal Cleavage
54	807.39	23.2	CLU	366–371	---	LNEQ| **FNWVSR** |LANL	---
1059	920.34	21.2	---	---	---	---	---
1160	928.51	24.6	UMOD	592–599	MEP1A, MMP3	RSGS| **VIDQSRVL** |NLGP	MEP1A, KLK6, CTS [B,D,E]
1778	981.49	24.4	ACTB	107–115	MEP1A, MMP [3,13], CTS [B,D,E]	HPVL **|LTEAPLNPK|** ANRE	---
2314	1032.45	25.9	ACTB	95–103	---	YNEL| **RVAPEEHPV** |LLTE	MEP1A, MMP [3,13], CTS [B,D,E]
3559	1134.59	24.0	COL3A1	755–766	---	ADGV| **PGKDGPRGPTGP** |IGPP	---
3662	1142.55	22.0	ADGRF3	56–64	---	DKAW| **NERIDRPFP** |ACPI	---
4564	1199.55	20.8	TKT	343–352	---	DGDT| **KNSTFSEIFK** |KEHP	---
4610	1201.53	24.9	GAGE12H	58–70	MEP1A, MMP3, CTSB	AAAQ| **KGEDEGASAGQGP** |KPEA	MMP3
4829	1217.48	27.7	COL3A1	179–191	---	PAGP| **pGPpGPpGTSGHp** |GSPG	KLK6, CTSB
5284	1250.64	20.6	HBB	136–147	---	QKVV| **AGVANALAHKYH**	*C-terminal end*
6601	1352.78	22.0	AHNAK	772–784	---	EVDV| **NLPKADVDISGPK** |IDVT	MEP1A, KLK6
6607	1353.53	23.8	FGA	605–617	MEP1A	YKMA| **DEAGSEADHEGTH** |STKR	---
8720	1513.62	29.5	CDH1	397–410	---	ITTL| **KVTDADAPNTPAWE** |AVYT	---
9080	1539.74	40.4	COL18A1	1400–1416	MEP1A, CTSB	EGRQ| **GPpGPpGPPGPPSFPGP** |HRQT	MMP3
9510	1576.68	44.9	---	---	---	---	---
9728	1594.77	40.3	COL1A1	1177–1194	MMP3	DAGP| **VGPpGPpGPpGPpGPPSA** |GFDF	MMP [3,13], CTSB
10177	1624.73	25.1	COL2A1	1150–1167	---	GPSG| **DQGASGpAGpSGpRGPpG** |PVGP	---
11725	1733.73	29.8	GSN	605–621	CTSD	AAYL| **WVGTGASEAEKTGAQEL** |LRVL	MEP1A, MMP3, CTS [D,E]
12459	1782.85	26.0	---	---	---	---	---
13134	1836.79	31.1	COL1A2	918–937	MMP3	SPGV| **NGApGEAGRDGNPGNDGPpG** |RDGQ	MEP1A, MMP [3,13], CTSB
13176	1840.83	41.9	COL2A1	1193–1213	MEP1A, KLK6	PRGR| **SGETGPAGppGNPGPPGPpGP** |PGPG	KLK6
14389	1930.89	31.6	COL3A1	618–639	MEP1A, CTSB	TGPQ| **GPpGPTGPGGDKGDTGPpGPQG** |LQGL	MEP1A, MMP [3,13], CTSB
14925	1972.96	25.0	PCSK1N	223–241	MEP1A	RRAA| **DHDVGSELPPEGVLGALLR** |VKRL	MEP1A, MMP [3,13]
15342	2009.88	32.4	COL5A2	136–157	MEP1A, MMP [3,13], CTSB	GAPG| **SKGEAGpTGPMGDpGTVGPPGP** |VGER	MMP3
17066	2169.98	33.7	COL16A1	1145–1166	MEP1A, MMP [3,13], CTSB	GPQG| **NSGEKGDQGFQGQPGFpGPPGP** |PGFP	---
17805	2232.00	33.6	COL5A1	999–1021	---	PPGV| **VGpQGpTGETGpMGERGHPGPpG|PPGE**	MEP1A, CTSB
19681	2380.08	35.8	COL3A1	201–228	MEP1A, CTSB	PGYQ| **GPPGEPGQAGpSGpPGppGAIGPSGPAG** |KDGE	MEP1A, MMP [3,13], CTSB
20237	2430.60	35.7	---	---	---	---	---
24328	2854.37	34.6	COL3A1	616–646	CTSB	GETG| **PQGPpGPTGpGGDKGDTGPpGPQGLQGLpGT** |GGPP	MEP1A, CTSB
29919	3524.75	32.4	CLU	390–423	---	VTTV| **ASHTSDSDVPSGVTEVVVKLFDSDPITVTVPVEV** |SRKN	MMP3
ACTB, Actin, cytoplasmic 1; ADGRF3, Adhesion G-protein coupled receptor F3; AHNAK, Neuroblast differentiation-associated protein; CDH1, Cadherin-1; CLU, Clusterin; COL1A1, Collagen α-1(I) chain; COL1A2, Collagen α-2(I) chain; COL2A1, Collagen α-1(II) chain; COL3A1, Collagen α-1(III) chain; COL5A1, Collagen α-1(V) chain COL5A2, Collagen α-2(V) chain; COL16A1, Collagen α-1(XVI) chain; COL18A1, Collagen α-1(XVIII) chain; CTS, Cathepsin; FGA, Fibrinogen α chain; GAGE12H, G antigen 12H; GSN, Gelsolin; HBB, Hemoglobin subunit β; KLK6, Kallikrein-6; MEP1A, Meprin A subunit α; MMP, Matrix metallopeptidase; PCSK1N, ProSAAS; TKT, Transketolase; UMOD, uromodulin.							

Abbreviation: AA, amino acid sequence; CE, capillary electrophoresis; Exp. mass, experimental mass. ^†^ Peptide identification number. ^‡^ Lower case *p* expresses hydroxyproline.

**Table 4:** Differences in the activities of the seven in silico predicted proteases meprin A subunit α (MEP1A), matrix metallopeptidase (MMP) 3 and 13, kallikrein-6 (KLK6) and cathepsin (CTS) B, D and E based on the fold change of the protease associated peptide substrate’s ion signals between the HCC case and non-HCC liver disease control groups. *p*-values were calculated by the Mann–Whitney U test.

Protease	Peptide Substrate Distribution [Avg. Ion Counts ± SD]		Fold-Change Case/Control	*p*
	HCC Case Group (*n* = 18)	Non-HCC Liver Disease Control Group (*n* = 51)		
MEP1A	196.23 ± 93.19	365.65 ± 231.12	0.54	0.003
MMP3	632.99 ± 317.56	393.63 ± 331.29	1.60	0.007
MMP13	729.36 ± 402.76	495.03 ± 539.78	1.47	0.012
KLK6	166.40 ± 79.87	67.09 ± 88.51	2.47	<0.0001
CTSB	347.63 ± 173.67	643.77 ± 399.10	0.53	0.004
CTSD	32.90 ± 33.44	17.35 ± 31.21	1.89	0.015
CTSE	34.41 ± 31.99	22.56 ± 41.25	1.52	0.031

**Table 5:** Allred Scoring for all retrieved tissue specimens in the study.

Tissue Type	Allred IHC Score	
	KLK6	MEP1A
HCC 1	3	0
HCC 2	3	1
HCC 3	3	0
HCC 4	3	0
HCC 5	3	0
Cirrhosis 1	2	0
Cirrhosis 2	2	0
Cirrhosis 3	3	0
Cirrhosis 4	3	0
Normal liver 1	1	1
Normal liver 2	1	0
Normal liver 3	1	1
Normal liver 4	1	1
Normal liver 5	1	1

**Table 6:** List of HCC-31 peptide markers that were identified in human tissue or body fluids other than urine.

HCC-31 Peptide Marker ID	Amino Acid Sequence	Protein Name	Protein Symbol	Biological Source of Identification (Other than Urine)	Reference
11354	107-LTEAPLNPK-115	Actin; α skeletal muscle	ACTA1	Cerebellum tissue ^‡^	Marcu et al. [33]
				HCC tumor tissue ^‡^	Liang-Qing et al. [34]
14071	95-RVAPEEHPV-103	Actin, cytoplasmic 1	ACTB	Lung tissue ^‡^	Marcu et al. [33]
25411	179-PGPPGPPGTSGHP-191	Collagen α-1(III) chain	COL3A1	Plasma	Zakharova et al. [35]
33901	605-DEAGSEADHEGTH-617	Fibrinogen α chain	FGA	Serum	Ueda et al. [36]
				Plasma	Koomen et al. [37]
135817	390-ASHTSDSDVPSGVTEV VVKLFDSDPITVTVPVEV-423	Clusterin	CLU	Cerebrospinal fluid	Belogurov et al. [38]
57312	605-WVGTGASEAEK TGAQEL-621	Gelsolin	GSN	Plasma	Modzdiak et al. [39]

^‡^ identified as a HLA-associated immunopeptide.

## Data Availability

All data are available in this manuscript and the open access database; Zenodo; https://zenodo.org/ and are linked to the DOI: 10.5281/zenodo.5138595, last accessed on 21 July 2021.

## Supplementary Materials

The following are available online at https://www.mdpi.com/article/10.3390/cancers13153786/s1, Supplementary Figure S1 graphical abstract. Supplementary Figure S2 flow chart showing the different phases of the study. Supplementary Figure S3 one-way ANOVA on subgroups of patients with NAFLD (*n* = 27), NASH without LC (*n* = 14), LC (*n* = 72) and HCC (*n* = 57) with respective Box-and-Whisker distribution plots. The supplementary material also contains details about steps taken to complete the immunohistochemistry.
