# Supplementary material for: Pathophysiological Implications of Urinary Peptides in Hepatocellular Carcinoma
Source: Cancers (Basel). 2021 Jul 27;13(15):3786. doi: 10.3390/cancers13153786 (PMC8345155; doi:10.3390/cancers13153786)
Supplement: Supplementary file 1 [file cancers-13-03786-s001.zip › cancers-1283735-supplementary.pdf]

## Figure S1. Graphical Abstract

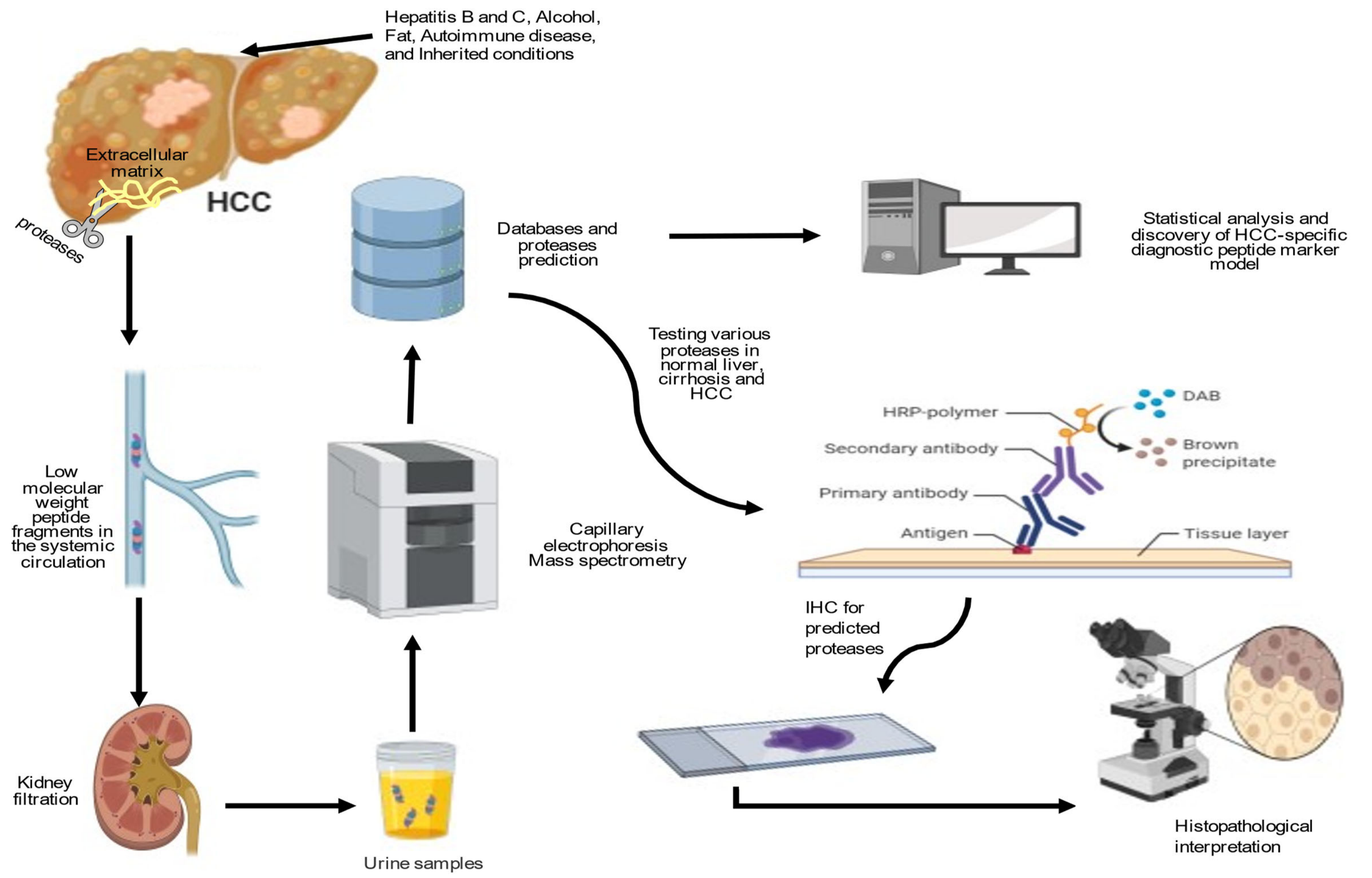


*Hepatocellular carcinoma (HCC), Horseradish peroxidase (HRP), Immunohistochemistry (IHC)

**Figure S2. Schematic study flow chart showing the different phases of the HCC study.**

## Discovery phase for peptide marker search and biomarker model establishment

18 HCC, 25 LC, 8 NASH w/o LC, 9 NAFLD, 9 center-matched NC

**1st peptide marker selection step:**
Statistical comparison of peptide amplitudes by a group-wise non-parametric Wilcoxon rank sum test with correction for multiple testing by the Benjamini & Hochberg procedure. → Extended list of 123 peptide marker

**2nd peptide marker selection step:**
Rank sum correlation of peptide amplitudes based on the level of liver damage (0 = NC/non-LC, 1 = LC, 2 = HCC). → Restricted list of 31 peptide marker

**SVM model generation:**
Integration of the 31 peptide markers to the SVM model HCC-31 and determination of a classification cut-off by ROC analysis.

## Validation phase for HCC-31 performance and prediction of patient outcome testing

39 HCC, 87 non-HCC liver diseases

**ROC analysis:**
AUROC of 0.88 (95% CI: 0.81-0.93, p<0.0001), and 79.5% sensitivity and 85.1% specificity at the predetermined cut-off at -0.25.

**Kaplan-Meier survival analysis:**
4.1-fold increased risk of death (95% CI: 1.7 – 9.8, p=0.0005) during the 500-days follow-up in case of a HCC-31 positive test result.

## Linkage of HCC-31 peptide markers to pathophysiological aspects of HCC

**Peptide sequencing:**
Amino acid sequence identification for 27 of the 31 peptides included in HCC-31.

**Protease prediction:**
Mapping of 7 proteases to the 31 peptides of HCC-31.

**IHC staining:**
Differential expression of KLK6 and MEP1A in liver tissue between HCC, LC and NC.

**List of Abbreviations:** AUROC, area under the ROC curve; CI, confidence interval; HCC, hepatocellular carcinoma; IHC, immunohistochemistry; KLK6, Kallikrein-6; LC, liver cirrhosis; MEP1A, Meprin A subunit $\alpha$; NAFLD, Non-alcoholic fatty liver disease; NASH, Non-Alcoholic Steatohepatitis; NC, normal controls; ROC, receiver operating characteristics; SVM, support vector machine.

**Figure S3. Box-and-Whisker distribution plot representations of clinical parameter levels demonstrating significant differences between HCC and non-HCC groups in Table 1 of the main manuscript further separated into NAFLD (N=27), NASH without LC (N=14), LC (N=72) and HCC (N=57) patient subgroups.** As revealed by this one-way ANOVA analysis, graduated differences in their levels exist, which relative to the HCC case group are more pronounced in patients without than in patients with LC manifestations. In fact, for most of these markers a substantial overlap between LC and HCC is evident.

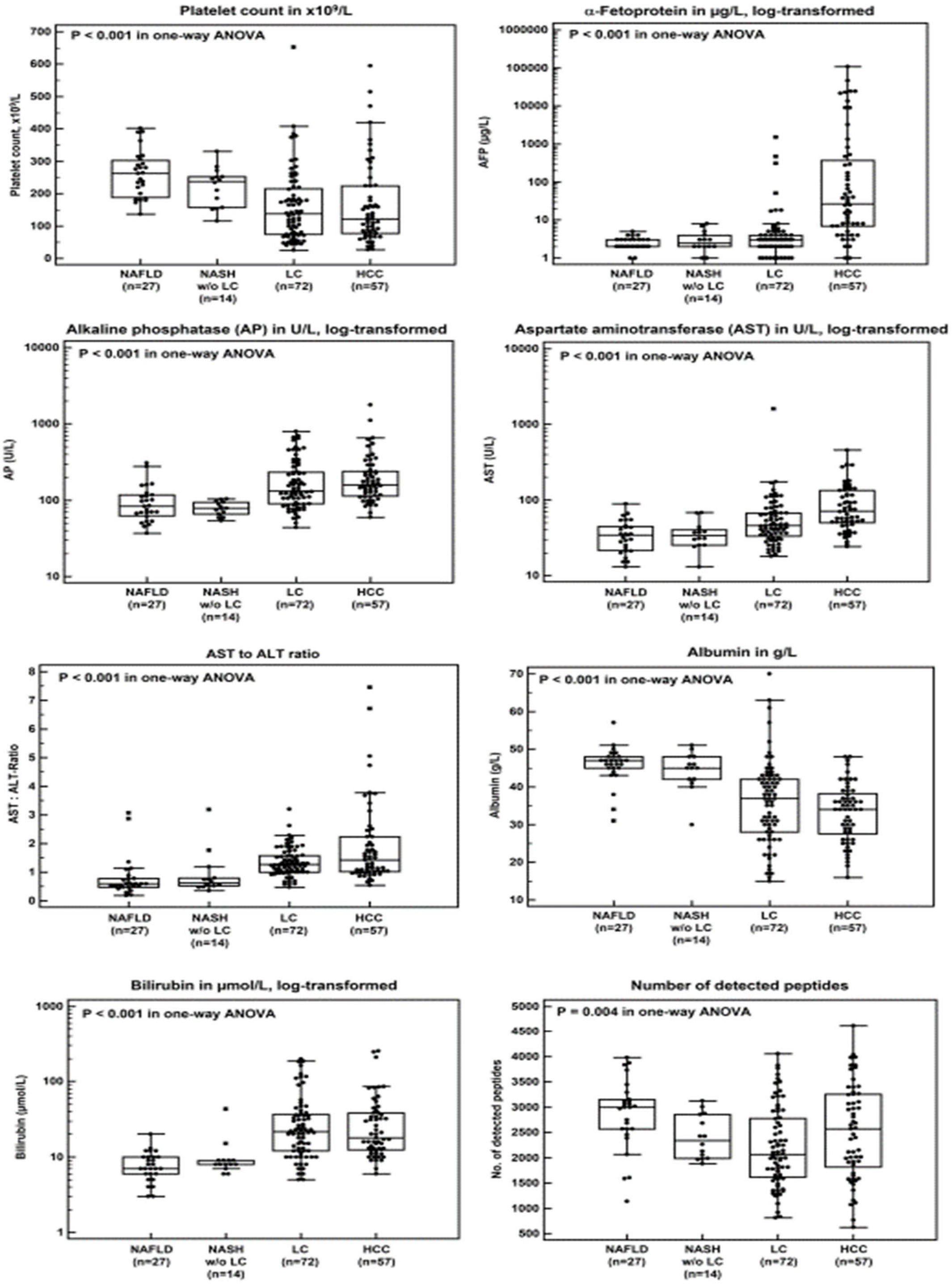

**Steps used in immunohistochemistry**

The tissue sections were formalin-fixed and paraffin-embedded using a standardized protocol. The tissue sections were cut at 3-4 µm and dried over-night in a 56 $^{o}$C oven. A Leica polymer detection kit (Leica, UK) was used throughout. Prelabelled slides were washed with distilled water. Endogenous peroxidase activity was blocked using 100 µl/2 drops of Leica peroxidase block and left for 5 minutes. Two washes were carried out using Tris-buffered saline (TBS) for five minutes each. Sections were then incubated in Leica protein block 100 µl for 20 mins; this reduces non-specific binding of primary and polymer. The protein block was drained off and a further two TBS washes were completed at five minutes each. Incubation in KLK6 at 1:200 and MEP1A at 1:1400 dilution was then performed as determined by prior serial dilutions. Incubation with antibody was performed overnight in the fridge at 4°C to help reduce the level of background staining. After overnight incubation, sections were washed in TBS for 10 minutes, twice. Leica post primary block was then applied to slides using 100µl and incubated for 30 minutes, this solution recognizes the goat immunoglobulins, this step is followed by two washes with TBS for 5 minutes each. Sections were then incubated in Leica Novo-link polymer solution using 100µl to ensure adequate coverage and incubated for 30 minutes followed by two washes with TBS for 5 minutes each time. Sections were incubated in Leica diaminobenzidine (DAB) working solution. This was constituted using 50µl of DAB chromogen in one ml of Novolink DAB substrate. Each slide was covered with two-three drops and left for five minutes. The reaction with the peroxidase produces a visible brown precipitate at the antigen site. This step was followed by a further two washes with TBS for five minutes each time. Slides were then rinsed in dis-tilled water and drained off. A counterstain of haematoxylin (purple colour) was ap-plied for 30 seconds and slides were again washed in distilled water, followed by incubation in TBS for 2-3 minutes. Once left to dehydrate they were then ready for viewing. Results were interpreted using a light microscope and Panoramic viewer (3DHISTECH Ltd, Hungary), where digitally converted slides were displayed.
